# Belzutifan for patients with Von Hippel-Lindau (VHL) disease-associated heterogeneous tumors – a retrospective single center analysis

**DOI:** 10.1186/s12885-025-15192-8

**Published:** 2025-11-01

**Authors:** Kilian Rhein, Fruzsina Kotsis, Athina Ganner, Sophie Astheimer, Christine Julia Gizaw, Yannik Laich, Cordula Annette Jilg, Jan-Helge Klingler, Elke Neumann-Haefelin

**Affiliations:** 1 Renal Division, Department of Medicine, Medical Center, Faculty of Medicine, University of Freiburg, University of Freiburg, Freiburg, Germany; 2 Eye Center, Faculty of Medicine, Medical Center - University of Freiburg, University of Freiburg, Freiburg, Germany; 3 Department of Urology, Faculty of Medicine, Medical Center - University of Freiburg, University of Freiburg, Freiburg, Germany; 4 Department of Neurosurgery, Faculty of Medicine, Medical Center - University of Freiburg, University of Freiburg, Freiburg, Germany; 5 Institute of Genetic Epidemiology, Faculty of Medicine, Medical Center - University of Freiburg, University of Freiburg, Freiburg, Germany; 6 Department II of Internal Medicine, Faculty of Medicine and University Hospital, University of Cologne, Cologne, Germany

**Keywords:** Von Hippel-Lindau disease, Belzutifan, HIF-2α inhibitor, Renal cell carcinoma, Hemangioblastoma, Neuroendocrine tumors, Targeted therapy

## Abstract

**Background:**

Von Hippel-Lindau (VHL) disease is a hereditary tumor predisposition syndrome characterized by benign and malignant tumors affecting multiple organ systems. Standard treatment primarily involves surgical resection and tumor ablation, which can lead to progressive organ dysfunction. Belzutifan, an oral hypoxia-inducible factor-2 alpha (HIF-2α) inhibitor, has emerged as a novel therapeutic option for VHL-associated tumors. However, data on its efficacy and safety outside of clinical trials remain limited. This study evaluates the outcomes of off-label belzutifan treatment in patients with advanced VHL-related organ manifestations, where standard therapeutic options were limited or contraindicated.

**Results:**

A retrospective analysis was conducted on eight patients with genetically or clinically confirmed VHL disease treated with belzutifan at a specialized VHL center in Germany. The median treatment duration was 16.4 months (range: 3.6–26 months). Disease stabilization was achieved in all patients, with a partial response observed in three cases. Tumor progression was noted in two patients, involving the emergence of new lesions, but no progression occurred in the primary target lesions, prompting treatment initiation. Belzutifan demonstrated tumor control across multiple organ systems, reducing the need for surgical interventions. The most common adverse event was anemia, occurring in all patients, with three requiring erythropoiesis-stimulating agents or dose reductions. Mild leukocytopenia and transient liver enzyme elevations were observed but did not require treatment modifications. Other adverse effects included infections, which were managed with temporary treatment interruptions.

**Conclusions:**

Belzutifan represents a promising treatment option for VHL disease, particularly in patients for whom surgery carries significant risks. The therapy demonstrated effective tumor control and delayed disease progression in most cases. However, anemia remains a key side effect requiring close monitoring and management. Larger, long-term studies are needed to further assess the efficacy and safety of belzutifan in VHL patients and to refine treatment strategies.

**Supplementary Information:**

The online version contains supplementary material available at 10.1186/s12885-025-15192-8.

## Background

Von Hippel-Lindau (VHL) disease (OMIM #193300) is a multisystemic tumor predisposition syndrome characterized by a diverse array of tumors, both benign and malignant, such as renal cell carcinoma (RCC), hemangioblastomas (HBL) of the central nervous system (CNS) and retina, pheochromocytomas (PHEO), paragangliomas (PGL), neuroendocrine tumors (NET), predominantly of pancreatic origin but also affecting the gastro-entero-pancreatic tract. Additional manifestations include endolymphatic sac tumors (ELST), cystadenomas of the epididymis, and cysts of the pancreas and kidneys [1]. The incidence of VHL is approximately 1 in 36,000, with a lifetime penetrance of 100% by age 75 [2]. The mortality associated with VHL disease is determined by complications arising from CNS HBL, RCC and NETs [3].

Surveillance and surgical resection or ablation are the primary approaches for most neoplasms associated with VHL [4, 5]. Patients with VHL disease have a lifelong risk of developing tumors in affected organs, and therefore most patients undergo multiple interventions throughout their lives. Even with cautious and parenchyma-sparing (micro-)surgery and interventions, functional outcomes may worsen, especially in advanced disease manifestations, e.g. for RCC, CNS HBL or central juxtapapillary retinal HBL [6–8]. To date, only a limited number of medical therapeutic strategies have been successfully implemented in VHL disease [9]. Therefore, alternative treatment options are urgently needed.

Insight into the pathogenesis of VHL disease has opened new targeted treatment options for VHL disease. Belzutifan is an oral small molecule inhibitor of hypoxia-inducible factor 2 alpha (HIF-2α) that disrupts HIF transcription complex formation and prevents downstream signaling and resultant oncogenesis [10–12]. Belzutifan was first evaluated in the non-randomized, phase II LITESPARK-004 study for patients with VHL-associated, localized RCC [13]. The demonstrated efficacy for RCC and non-RCC lesions led to the US FDA’s approval of Belzutifan in 2021 for the treatment of adult patients with VHL disease [9].

Belzutifan was used off-label in Germany until its marketing authorization in February 2025, with limited experience outside clinical trials and significant prescription costs, highlighting the need for further data on its safety and efficacy [14]. Over the past 30 years, substantial experience has been gained in the surveillance and treatment of VHL patients at the multidisciplinary Freiburg VHL Center, providing a comprehensive approach in managing this complex condition.

The aim of this study is to provide our experience on off-label belzutifan treatment in patients with advanced VHL-associated tumors, where existing therapies, according to clinical guidelines, posed a risk of compromising organ function or were ineffective.

## Methods

### Patients

The Freiburg VHL Center has provided care for VHL patients since 1992 and is one of the largest centers in Germany. Our center currently provides active care for at least 500 patients, with approximately 300 patients seen each year. In accordance with current international recommendations [15], our surveillance protocols include annual imaging of the abdomen and CNS, typically via magnetic resonance imaging, along with hormone level measurements (plasma metanephrines) and eye examinations.

Surveillance, both prior to and during treatment with belzutifan was conducted at the Freiburg VHL Center in collaboration with the relevant specialist departments. Written informed consent was obtained from all patients included in our retrospective analysis, which was approved by the responsible ethics committee of the University of Freiburg (approval number 79/20).

### Data collection

Data were collected from medical records. The inclusion criteria for the study were as follows: patients had to be over 18 years of age, have clinically or genetically confirmed VHL disease, and have been treated with belzutifan at a therapeutic dosage of 80–120 mg for at least two months between 2022 and 2024. Data collection encompassed a period starting up to three years prior to the initiation of belzutifan treatment and continued until the last follow-up consultation. The data collection period concluded on October 1, 2024.

### Treatment with Belzutifan

In contrast to the initial FDA approval of belzutifan in 2021 for treating VHL-related tumors (RCC, CNS HBL and pNET) that did not require immediate intervention, the indication for belzutifan in this study was based on advanced organ manifestations that necessitated timely intervention. This provided the basis for submitting applications for off-label therapy to health insurance funds. The decision to initiate belzutifan therapy was made on a case-by-case basis by a multidisciplinary group of VHL disease experts. Each patient underwent a comprehensive and individual assessment by a multidisciplinary team of experts. This assessment carefully considered organ involvement, tumor burden, prior treatments, and the patient’s overall clinical status. For most patients, conventional surgical procedures or tumor ablative techniques, including total or partial nephrectomy as well as brachytherapy, were associated with a high risk of loss of critical organ function. In particular, patients with extensive RCC manifestations and multiple prior interventions had severely limited functional reserve and faced a high likelihood that repeated nephrectomy would result in renal failure, necessitating renal replacement therapy. In patients with parapapillary hemangioblastomas, the threat of irreversible vision loss rendered ocular surgery clinically unfavorable. Additionally, platinum-based systemic chemotherapy, representing the standard of care in one particular case of metastatic neuroendocrine tumor, proved ineffective in controlling disease progression [16]. In one case, a patient declined surgical intervention despite its theoretical feasibility. Although formal quality-of-life assessment tools were not used, the anticipated impact on quality of life, particularly related to preserving vision and renal function, was a central part of the multidisciplinary deliberations. In all cases, the severity of organ dysfunction, lack of effective alternatives, and urgency of intervention led to the classification of belzutifan as a last-line, organ-preserving therapeutic option. Belzutifan was administered on an off-label basis, requiring approval from medical insurers on a case-by-case basis. Once insurance approval was obtained, treatment commenced with an initial dosage of 120 mg.

The surveillance of belzutifan treatment was conducted based on published protocols [13]. This included control examinations at our VHL center every three months, along with biweekly monitoring of laboratory parameters by their primary care physicians. The monitored parameters included blood count, liver enzymes (AST, ALT, bilirubin, INR), blood sugar/HbA1c, serum creatinine, serum urea, oxygen saturation, and urine dipstick tests during the first week after treatment initiation and up to six months thereafter. Based on the occurrence of side effects during the initial six months, the frequency of monitoring was adjusted to monthly intervals. Test results were shared with the VHL center and evaluated by physicians experienced in belzutifan therapy, who provided recommendations regarding necessary adjustments and control intervals. When undesirable drug effects were detected, surveillance was intensified, and adjustments, such as dose reduction or discontinuation of belzutifan, were made. In cases of moderate anemia, treatment with erythropoiesis-stimulating agents (ESA) was initiated.

Imaging studies to monitor tumor progression were performed at 3, 6, 9, and 12 months after treatment initiation, with follow-up intervals extended to every 3–6 months based on cost approval by insurers (ranging from 3 to 12 months). Individual neuroradiologic screening intervals were adjusted to allow for close monitoring of existing cysts or cyst development based on patient response and neurosurgical recommendations. Additionally, the reported risk of belzutifan-related intracranial hemorrhage was taken into consideration [17] and patients were informed accordingly. Deviations from the recommended intervals occurred for patients who had imaging outside the center or due to individual availability.

### Outcome assessment

Target lesions were defined in organs affected by VHL, with a target lesion being defined as either the primary reason for initiating belzutifan treatment in a patient or an organ lesion measuring over 10 mm in its largest diameter. Up to five lesions per affected organ system, meeting the mentioned criteria, were compiled to target lesions. Non-target lesions included smaller residual lesions within the organ or new lesions that appeared after the initiation of treatment. Lesions were identified based on radiologic imaging annotations and fundoscopy. Baseline measurements of the lesions were taken from the last available follow-up data before starting treatment. For patients with retinal HBL, comprehensive imaging was not consistently available, as many were initially treated at other primary centers and only referred to our institution for systemic therapy. In two patients with parapapillary HBL, tumor area was measured on pseudocolor widefield imaging (OPTOS, P200DTx) by two independent graders outlining the tumor area including surrounding subretinal fluid and hard exsudates. For other tumors, lesion size were extracted from radiologic reports and assessed independently by two blinded reviewers.

Treatment response was assessed for all monitored VHL manifestations in the patients after treatment initiation, based on clinical and imaging data. Treatment response was assessed according to the measured tumor reduction rate, which corresponds to the relative change in the largest lesion diameter compared to the baseline measurement. Tumor response was categorized as partial response (PR), stable disease (SD), or progressive disease (PD), in accordance with RECIST 1.1 criteria for individual organ manifestations [18]. Additionally, new lesions or the necessity for tumor reduction procedures were also defined as disease progression.

Treatment response was graphically represented for individual patients using a combined bar and line chart, illustrating dosage and tumor size progression for measurable organ lesions. Additionally, swimmer plots were used to depict organ-specific tumor response for all monitored lesions per patient, as well as tumor reduction procedures that occurred during belzutifan treatment. Due to the small and heterogeneous nature of this cohort, no statistical analysis was performed.

### Safety assessment

Data on adverse events was collected from patient records at our VHL center during on-site visits, as well as from primary care physician records sent to our center. The data included laboratory results and assessments of the patients’ subjective well-being. The reference range provided by the laboratories was used to assess adverse effects. The severity of anemia was assessed according to the National Cancer Institute Anemia Scale [19].

## Results

### Patient characteristics

**Fig. 1:**
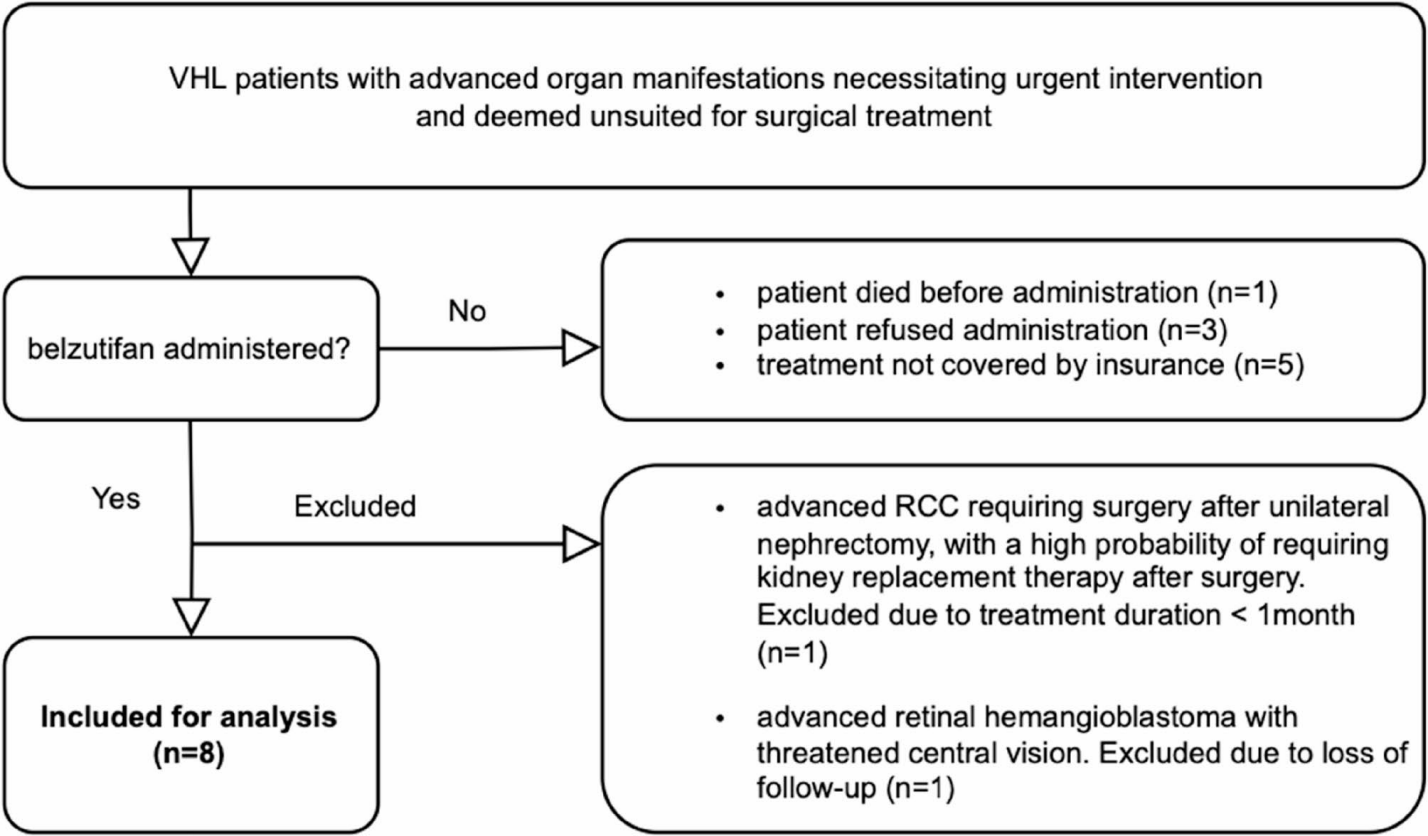
Flowchart of patient selection for evaluation of tumor response and safety under belzutifan in patients with VHL disease at a specialized tertiary medical center. The chart outlines the inclusion and exclusion criteria, resulting in the selection of eight patients for analysis, with patients denied treatment by insurers and one with a short treatment duration serving as internal controls for tumor progression

To assess the efficacy and safety of belzutifan, we conducted a retrospective study on patients with advanced VHL-related organ manifestations treated at our specialized VHL center. The patient selection process is detailed in the flowchart (Fig. 1).

Following eligibility screening, ten patients with genetically or clinically diagnosed VHL disease were enrolled in the retrospective analysis. These patients presented with conditions including advanced RCC, metastasized NET, HBL of the CNS and advanced retinal HBL. However, one patient was excluded due to a treatment duration of less than one month, and another was excluded due to loss of follow-up.

Consequently, eight patients with VHL disease, as detailed in Table 1 and Supplementary Table 1, were included in the final analysis. The median age at the initiation of belzutifan treatment was 45 years, with a range of 29 to 59 years. The median age at VHL diagnosis was 27 years, with a range of 18 to 41 years. The cohort comprised five males (63%) and three females (37%), all diagnosed with subtype I VHL disease [20]. Prior to belzutifan initiation, all patients displayed CNS HBL, 88% had retinal HBL, 75% had RCC, and 50% had NET of the gastrointestinal tract, including one patient with gastric NET and three with pancreatic NET. Overall, seven patients (88%) had undergone previous surgeries or ablative procedures, with a median of 13 procedures per patient and a range of 0 to 25.

**Table 1:** Baseline descriptive data on patients receiving Belzutifan

Patient characteristics	All Patients (*n* = 8)
Age — yr	
At time of belzutifan treatment beginning — median [range]	45 [29–59]
At time of VHL disease diagnosis — median [range]	27 [18–41]
Sex — no. (%)	
Male	5 (63)
Female	3 (37)
VHL disease subtype — no. (%)	
I	8 (100)
*VHL* mutations	
Arg167Trp, Exon 1 deletion, Exon 3 deletion, Gln164Leu, Ser80Arg, 527del Gly, genetic testing result not available in one patient^a^	
VHL manifestations — no. (%)	
Retinal hemangioblastomas	7 (88)
CNS hemangioblastomas	8 (100)
Renal cell carcinoma	6 (75)
Neuroendocrine tumors gastrointestinal tract	4 (50)
Pheochromocytoma	0 (0)
Metastatic disease	1 (13)
Previous surgery or ablative procedure — no. of patients (%) [range per organ]	
Any	7 (88) [0–25]
For renal cell carcinoma	6 (75) [0–6]
For CNS hemangioblastoma	7 (88) [0–11]
Pancreas-related surgery	2 (25) [0–1]
For pheocromocytoma	1^b^ (13) [0–1]
For retinal hemangioblastoma	7 (88) [0–17]
Previous surgical or ablative procedures per patient — median	13

^a^: clinically diagnosed patient

^b^: adenoma on histology

### Tumor response under belzutifan treatment

**Fig. 2:**
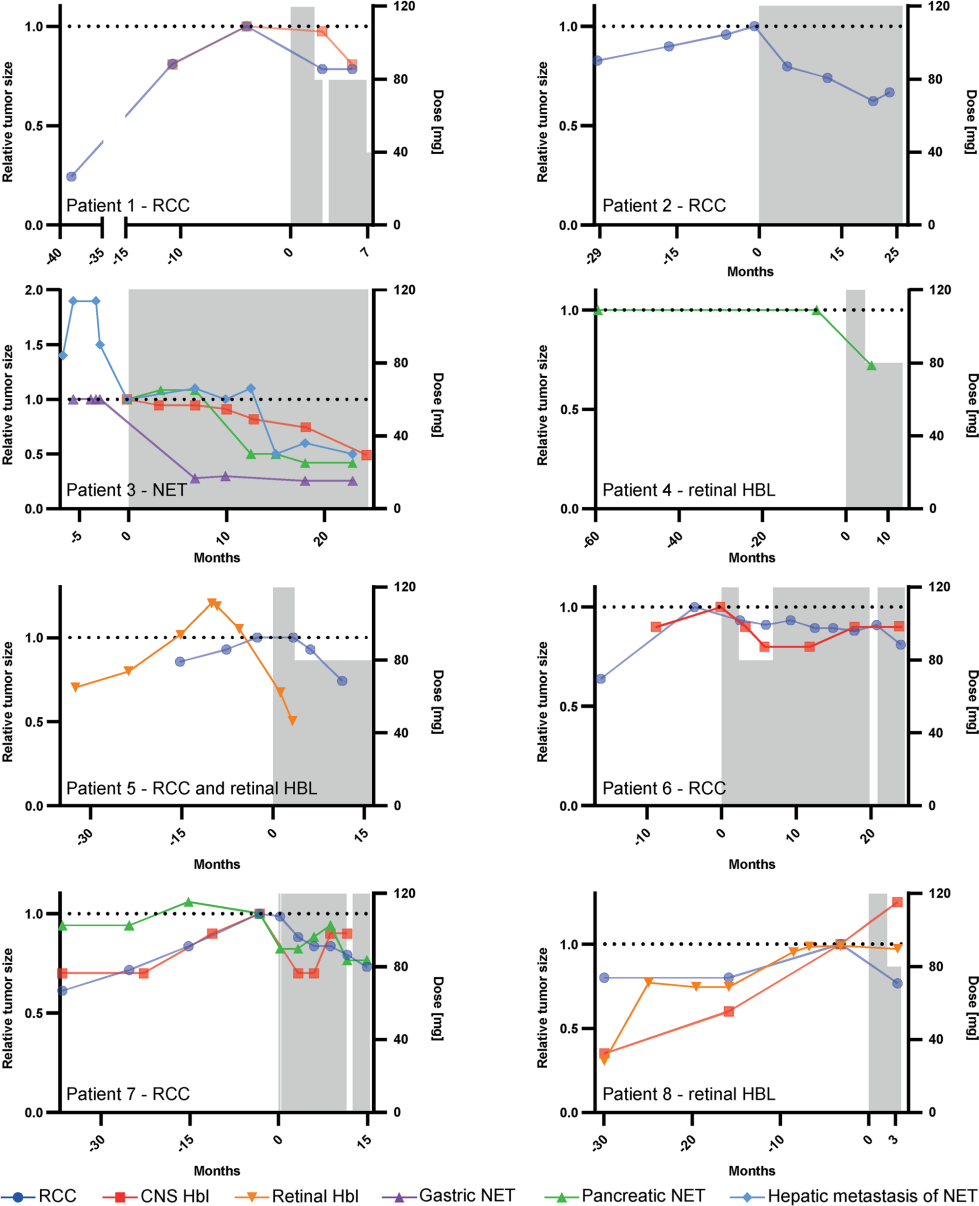
Relative tumor size tumor size over time during belzutifan treatment. Tumor size of measurable lesions is shown as a percentage of baseline (pre-treatment measurement) with concurrent belzutifan dosage indicated over the treatment period. Treatment response for organ lesions not measured (small punctiform CNS HBL or retinal HBL clinically followed) was graphically depicted in the swimmer plot in Fig. 3. The organ manifestation leading to treatment with Belzutifan is specified individually for each patient in the graph

The median treatment duration until the end of data collection was 16.4 months (range 3.6 to 26 months). Treatment interruptions occurred in three patients: Patient 1 experienced a two-week interruption due to anemia; Patient 6 had a six-week delay due to delayed reapproval by the insurer; Patient 7 had a one-week interruption due to an infection, followed by a two-week interruption due to low-grade anemia prior to elective surgery. Treatment with belzutifan led to a reduction in tumor size progression for most organ manifestations across all patients, as shown in Fig. 2.

**Fig. 3:**
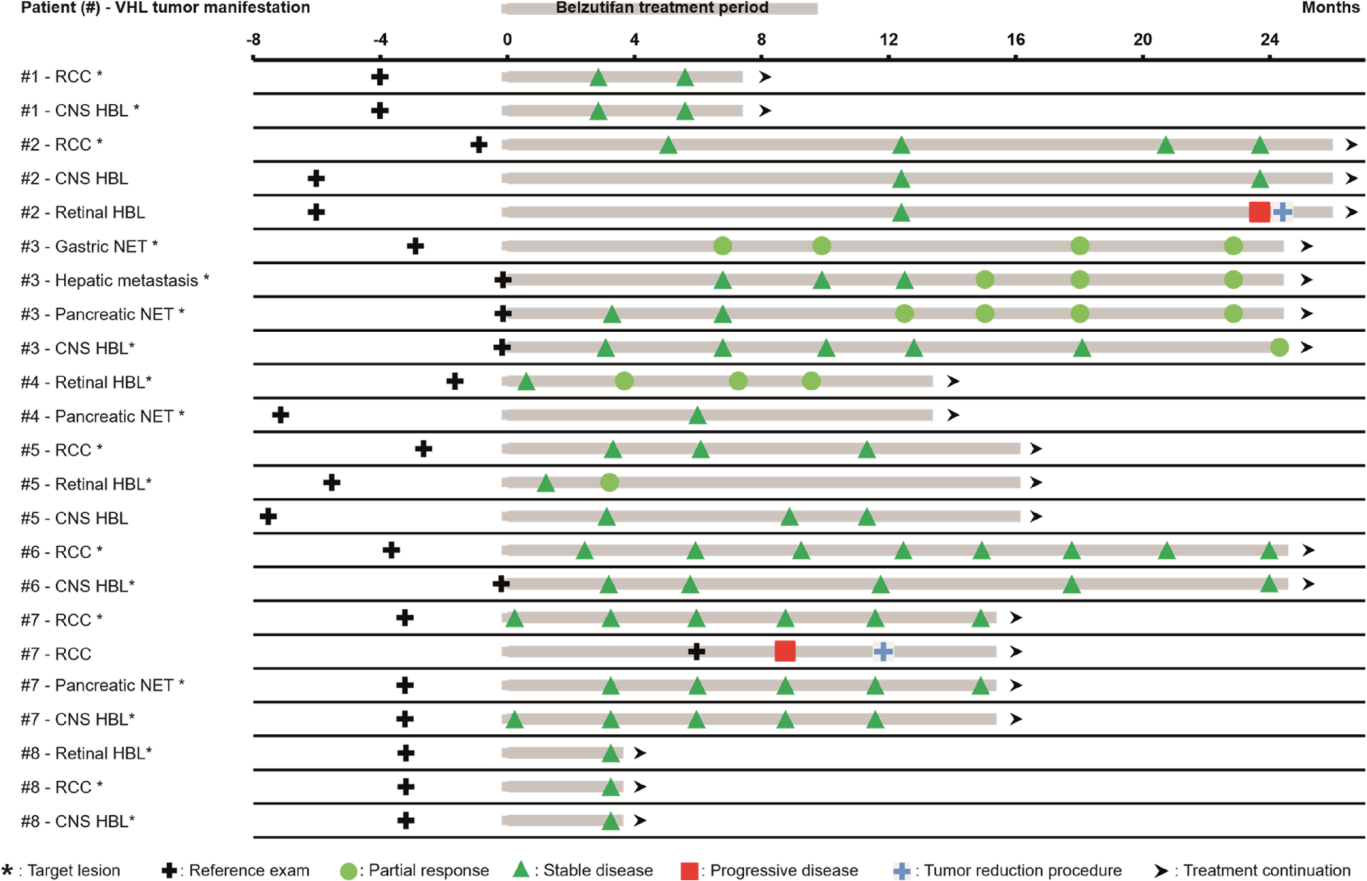
Swimmer plot of tumor response during belzutifan treatment Tumor response is shown over the treatment period (in months) for patients #1 - #8 treated with belzutifan. Each row represents monitored target and non-target lesions for individual patients

Overall, when considering all investigated organ sites, including target and non-target lesions, disease stabilization in at least one affected organ was achieved in all patients (Fig. 3). A partial response was observed in three patients. Disease progression was noted in two patients: one patient developed a new small retinal hemangioblastoma after 23.6 months of treatment, which was treated with focal laser coagulation. The other experienced a recurrence of renal cell carcinoma at the nephrectomy site after 11 months of treatment, requiring surgical intervention. Despite the emergence of new lesions in these two cases, importantly, no progression was observed in the initially affected lesions that led to the initiation of belzutifan treatment. In contrast, patients selected by the interdisciplinary VHL expert team for belzutifan treatment but denied coverage by insurers (4 out of 5 cases) or who stopped treatment after a few days of treatment for non-medical reasons (1 out of 5 cases) had higher rates of tumor progression (4 out of 5 cases) and a greater need for tumor reduction procedures (4 out of 5 cases) (supplementary Fig. 1).

The most significant decrease in tumor size was observed in the first 12 months of treatment (Fig. 2 and Supplementary Table 2). Overall, tumor regression was observed across all affected organs when considering the entire study period.

In one patient with a gastric neuroendocrine tumor (NET) and subsequent hepatic metastases, belzutifan treatment appeared particularly effective. Initial diagnosis via esophagogastroduodenoscopy (EGD) and targeted biopsy confirmed a grade 3 gastric NET. The patient received four cycles of etoposide combined with platinum-based chemotherapy over three months; however, this regimen failed to produce a radiologic response or reduce serum chromogranin A levels. Consequently, treatment was switched to belzutifan. At 21 months following initiation of belzutifan, follow-up EGD and biopsy revealed residual gastric NET downgraded from G3 to G1, indicating a marked improvement in tumor differentiation over the course of belzutifan therapy. An effect of prior chemotherapy on tumor differentiation, rather than tumor size, cannot be excluded and may have contributed to the observed grade reduction. Although the sequential biopsy was obtained from the same mass, sampling bias due to intratumoral heterogeneity cannot be ruled out.

All patients continued belzutifan treatment after data collecting ended.

### Safety

All patients exhibited signs of minor adverse events (Table 2). Anemia, an expected adverse event due to the HIF-2α inhibitory effects of belzutifan, was observed in all patients. Anemia-related symptoms, such as fatigue, were reported in two cases. In this cohort, therapy modifications were made based on clinical presentation rather than fixed hemoglobin thresholds. These patients showed a hemoglobin decline of approximately 28% compared to baseline. Symptomatic anemia prompted active intervention, while mild, asymptomatic hemoglobin declines were observed without treatment changes. Interventions included first reducing the dose of belzutifan from 120 mg once daily to 80 mg followed by administration of the erythropoiesis-stimulating agent darbepoetin-α 20 µg if hemoglobin levels continued to decrease or remained low after dose reduction. ESA administration occurred in three patients, either weekly or every other week, with close monitoring of hemoglobin levels and symptom resolution. Permanent belzutifan dose reduction was necessary in three patients. Darbepoetin-α dosing and treatment intervals were subsequently adjusted according to hemoglobin response and clinical improvement. Interestingly, one patient was able to return to the full dose of 120 mg after 4.5 months of dose reduction. No protocol beyond standard ESA dosing practices was applied.

**Table 2:** Adverse events observed under Belzutifan treatment

Adverse event — no. (%)	Population (*n* = 8)
Serious adverse event ¶	0 (0)
Minor adverse event	8 (100)
Self-reported symptoms	
Mild	2 (25) - fatigue and exertion fatigue
Moderate	0 (0)
Severe	0 (0)
Mild Leukopenia ◊	5 (63)
Transiently elevated transaminases	4 (50)
Anemia ✢	
Mild	4 (50)
Moderate	4 (50)
Severe	0 (0)
Infection ✦	2 (25)
New-onset diabetes mellitus	2 (25)
Resulted in	
Treatment interruption	1 (13)
Dose reduction	5 (63)
Treatment discontinuation	0 (0)

¶ A serious adverse event was defined as an event of any severity that was associated with permanent discontinuation of belzutifan

◊ Diagnostic observation only - no intervention indicated

✢ Three patients were administered erythropoiesis stimulating agents

✦ Herpes zoster after first week of treatment in one patient and persistent mild respiratory infection of the upper respiratory tract

Liver toxicity was monitored by screening for transaminase elevation. We observed mild elevations in four patients, with levels remaining below three times the upper normal limit. These elevations were transient and resolved within a few weeks. Importantly, no patients exhibited clinically corresponding symptoms, such as jaundice, and there were no instances where treatment discontinuation or dose reduction was necessary due to liver toxicity.

In five patients (63%), a transient or permanent reduction in total white blood cell counts was observed. Leukocytopenia was mild and did not result in treatment interruption or subjective symptoms. Two patients experienced infections, unrelated to leukocytopenia, while on belzutifan that necessitated treatment modifications: one patient developed herpes zoster on the arm, leading to a one-week treatment interruption, while another had a persistent mild upper respiratory infection accompanied by anemia, resulting in a dose reduction.

Two previously pre-diabetic patients experienced an increase in HbA1c levels meeting the diagnostic criteria for new-onset diabetes mellitus, accompanied by weight gain during belzutifan treatment.

## Discussion

Our study evaluated the efficacy and safety of belzutifan in eight patients with VHL disease. The findings from this study demonstrate that belzutifan is an effective treatment for managing advanced manifestations of VHL disease. Disease stabilization was observed in at least one affected organ in all patients, with a partial response achieved in three cases. These findings are in line with previously reported outcomes that showed an overall response rate of 49% and disease stabilization in another 49% for patients with VHL-associated renal cancer [13]. And overall response rates ranging from 30 to 90%, across other tumor entities [13]. Despite the emergence of new lesions in two cases under belzutifan, one retinal hemangioblastoma and a recurrence of renal cell carcinoma, the stabilization of initially affected lesions that led to the initiation of treatment indicates that belzutifan can effectively contain existing disease, even in metastatic status. Although our data are anecdotal and descriptive, they describe accelerated disease progression and increased procedural burden among patients who were denied insurance coverage and therefore did not recieve belzutifan. To rigorously assess its real-world efficacy, matched-control studies are warranted.

Our results underscore belzutifans´ ability to control tumor growth across multiple organ systems, providing a much-needed therapeutic option, especially for patients who may not be candidates for further surgical interventions. Thereby potentially delaying or preventing organ function loss due to intervention or disease progression. The noted disease progression emphasizes the importance of maintaining tumor surveillance protocol intervals, including regular imaging and funduscopic examinations.

While the effectiveness of belzutifan in this cohort is promising, the treatment does present some challenges due to its side effect profile. Anemia, a known consequence of HIF-2α inhibition, was observed in all patients, with varying degrees of severity but particularly pronounced in female patients. Moderate anemia required the use of erythropoiesis-stimulating agents in three patients, and dose reductions were necessary in three cases. It is important to reassess dose reductions considering the noted adaptations following initial declines in hemoglobin levels, which showed slight improvement throughout the treatment course. The observed rates are slightly higher compared with findings from larger studies [13, 21] that described anemia rates of 83 and 90% respectively. The slightly higher incidence in our series—without an increase in high-grade events—is most likely attributable to our small sample size. These findings indicate that anemia remains a manageable but significant side effect that requires regular monitoring and individualized treatment plans. Major trials of belzutifan in von Hippel–Lindau disease and renal cell carcinoma allowed concomitant ESA use for symptomatic anemia. No signal emerged that ESAs compromised tumor response rates or progression-free survival [13, 21]. Transient elevations in liver enzymes and leukocyte count decrease were also noted in four and five patients, respectively, but did not result in clinically significant symptoms or the need for dose adjustments. Infections emerged as another concern with temporary treatment interruption in one patient. This highlights the need for a proactive approach in managing infections and other potential adverse effects through close monitoring of patients undergoing belzutifan treatment. Currently, there are no established recommendations regarding specific pre-treatment immunizations for patients initiating belzutifan therapy, and such measures are not routinely practiced at our institution. In larger clinical trials, upper respiratory tract and urinary tract infections were among the frequently reported infectious adverse events; however, no consistent evidence has indicated an increased infection risk that would justify specific preventive vaccination [13, 21]. While the occurrence of viral infections in our cohort may justify discussing vaccination status during individualized patient counseling, at present we do not see sufficient evidence to support routine pre-treatment immunizations beyond standard recommended vaccinations for patients undergoing belzutifan therapy.

Regular check-ups and laboratory assessments should be standard practice to detect any emerging adverse events early. Additionally, patient and local primary care provider education plays a key role in the early identification of serious side effects, allowing for prompt intervention and minimizing disruptions to treatment.

The initiation of belzutifan treatment should be guided by a decision made by a multidisciplinary team experienced in managing VHL disease [22]. This team should include specialists from internal medicine, urology, radiology, neurosurgery, and ophthalmology. As VHL patients display large phenotypic variation [23], patients must be risk-stratified individually. Continuous evaluation of treatment response and disease progression should be conducted at a specialized VHL center. Our study employed regular imaging and clinical evaluations, which were essential in guiding decisions about dose adjustments and the need for supplementary treatments.

This study presents several significant strengths that enhance its contributions to the understanding of belzutifan’s efficacy and safety in treating advanced VHL-associated tumors. Firstly, the research is based on real-world data and, to our knowledge, represents the largest cohort of VHL patients, offering valuable insights into the use of Belzutifan, particularly given the limited clinical trial data for this patient population and its wider availability following approval in an increasing number of countries. Conducted at the Freiburg VHL Center, one of Germany’s largest and most experienced institutions for VHL management, the study benefits from the expertise of a multidisciplinary team familiar with the complexities of VHL disease. The center’s thorough patient monitoring protocol, including regular imaging and laboratory assessments, ensures accurate evaluations of treatment responses and potential adverse effects. Furthermore, the inclusion of a diverse cohort with various advanced manifestations of VHL broadens the applicability of the findings across different tumor types. Notably, the FDA approval of belzutifan for renal cell carcinoma signifies its promising potential for broader use, extending beyond the VHL patient population [21]. This approval underscores the clinical relevance of the study’s findings for a wider range of patients. Additionally, the documentation of the safety profile of belzutifan provides crucial information regarding its adverse effects, particularly anemia. Together, these strengths highlight the study’s important role in advancing the treatment of VHL and its potential to influence clinical practice.

This study has several limitations that should be considered. First, the inclusion of eight patients in the final analysis limits the generalizability of the findings to a broader population of individuals with VHL disease. While the heterogeneous nature of the cohort reflects the clinical complexity of VHL management, it makes it challenging to draw definitive conclusions about the drug’s efficacy across specific types of VHL-related tumors. Establishing multicenter registries or collaborative patient cohorts will be critical to assemble larger, more diverse populations and validate our preliminary observations. The availability of belzutifan also posed a challenge in the study. As the drug was administered on an off-label basis, securing insurance approval was necessary for each case. Consequently, the study may not fully reflect the potential effectiveness and safety profile of belzutifan across the broader VHL patient community. These limitations highlight the need for future studies in this patient population that can include a larger and more representative sample.

Despite these limitations, the study provides valuable insights into the real-world use of belzutifan for managing complex manifestations of VHL disease, offering a basis for further research and clinical evaluation. While this study did not specifically assess patient-based outcomes, such as quality of life, future research could benefit from including these measures to better balance between long-term drug therapy and intermittent surgical interventions and thereby lead to more personalized treatment decisions for the broader VHL population. Additionally, further research is needed to better identify which patients are at greater risk of disease progression and would benefit most from belzutifan therapy. The decision on treatment indication should be guided by individual progression risk, incorporating clinical and genetic data.

## Conclusion

In conclusion, belzutifan represents a promising and safe therapeutic option for patients with VHL disease, particularly when surgical interventions are not feasible or carry high risks. Its ability to stabilize disease across multiple organ systems makes it valuable in the management of this complex condition. However, its administration requires a well-coordinated approach involving close monitoring, regular assessments, and a multidisciplinary team to address the complexities of both the disease and the treatment. Future studies with larger patient cohorts and longer follow-up periods will be important to further clarify the long-term efficacy and safety of belzutifan and to establish guidelines for its use in clinical practice. Collaboration between specialized centers, primary care providers, and patients will be essential to maximize the benefits of this therapy and improve outcomes for those affected by VHL disease.

## Supplementary Information

Supplementary Material 1.

Supplementary Material 2.

Supplementary Material 3.

## Data Availability

Public posting of individual level participant data is not covered by the informed patient consent form. The datasets generated and analysed during the current study are not publicly available to respect participants’ rights to privacy and to protect their identity but are available from the corresponding author on reasonable request. Upon approval of a scientific project proposal, collaborating scientists may receive a dataset containing pseudonyms, as indicated in the patient’s informed consent form and as approved by the ethics committees.

## Abbreviations

ALT: Alanine aminotransferase
AST: Aspartate aminotransferase
CNS: Central nervous system
ELST: Endolymphatic sac tumor
EGD: Esophagogastroduodenoscopy
ESA: Erythropoiesis–stimulating agent
FDA: Food and drug administration
G1, G3: Tumor grading levels
HbA1c: Glycated hemoglobin
HBL: Hemangioblastoma
HIF: 2α–Hypoxia–inducible factor–2 alpha
INR: International normalized ratio
NET: Neuroendocrine tumor
OMIM: Online mendelian inheritance in man
PGL: Paraganglioma
PHEO: Pheochromocytoma
PR: Partial response
PD: Progressive disease
pNET: Pancreatic neuroendocrine tumor
RCC: Renal cell carcinoma
RECIST: Response evaluation criteria in solid tumors
SD: Stable disease
VHL: Von Hippel–Lindau
