# Supplementary material for: Belzutifan for patients with Von Hippel-Lindau (VHL) disease-associated heterogeneous tumors – a retrospective single center analysis
Source: BMC Cancer. 2025 Nov 1;25:1686. doi: 10.1186/s12885-025-15192-8 (PMC12579424; doi:10.1186/s12885-025-15192-8)
Supplement: Supplementary file 1 — Supplementary Material 1. [file 12885_2025_15192_MOESM1_ESM.pdf]

Supplementary Figure 1:

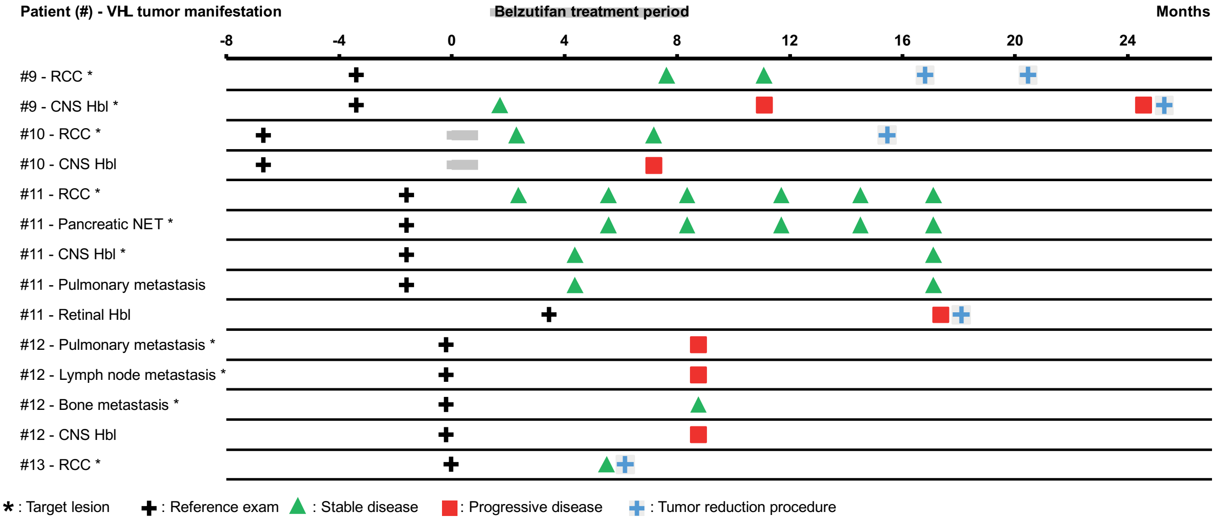

Supplementary Figure 1: *Swimmer plot of tumor response for patients selected by the interdisciplinary VHL expert team for belzutifan treatment but denied coverage by insurers (4 out of 5 cases) or who stopped treatment after a few days of treatment for non-medical reasons (1 out of 5 cases)*

Each row represents monitored target and non-target lesions for individual patients.
