# Supplementary material for: Belzutifan for patients with Von Hippel-Lindau (VHL) disease-associated heterogeneous tumors – a retrospective single center analysis
Source: BMC Cancer. 2025 Nov 1;25:1686. doi: 10.1186/s12885-025-15192-8 (PMC12579424; doi:10.1186/s12885-025-15192-8)
Supplement: Supplementary file 2 — Supplementary Material 2. [file 12885_2025_15192_MOESM2_ESM.pdf]

Supplementary Table 1:

Supplementary Table 1. Individual patient characteristics, VHL mutations, prior interventions, reasons for initiating belzutifan treatment, treatment duration, best tumor response, adverse events, and treatment modifications. Abbreviations: VHL, von Hippel-Lindau; HBL, hemangioblastoma; RCC, renal cell carcinoma; CNS, central nervous system; NET, neuroendocrine tumor; ALT, alanine aminotransferase; AST, aspartate aminotransferase; ESA, erythropoiesis-stimulating agent; SD, stable disease; PR, partial response.

Patient (# 1-8)		#1	#2	#3	#4	#5	#6	#7	#8
Sex		female	male	male	female	male	male	male	female
VHL Mutation		238 A>C (S80R)	genetic testing result not available	Del Exon 1	499C>T (Arg167Trp)	491A>T (Q164L)	Del Exon 3	527 del G (Frameshift)	Del Exon 3
Age at first manifestation		27	18	36	31	41	26	27	21
VHL manifestations prior to belzutifan treatment		Retinal HBL, CNS HBL, RCC, pancreatic NET	Retinal HBL, CNS HBL, RCC	CNS HBL, RCC	Retinal HBL, CNS HBL, pancreatic NET	Retinal HBL, CNS HBL, RCC	Retinal HBL, CNS HBL, RCC	Retinal HBL, CNS HBL, RCC, pancreatic NET	Retinal HBL, CNS HBL, RCC
Tumor reduction interventions or systemic treatment prior to belzutifan treatment for:	Retinal HBL	1	9	0	13	17	1	2	5
	CNS HBL	7	2	0	7	2	6	2	11
	RCC	5	1	0	0	6	2	6	1
	NET	1	0	0	1	0	0	0	0
	other	0	0	4 cycles of etoposide/platin based chemotherapy	1 (adrenalectomy for pheocromocytoma - adenoma on histology)	0	0	0	0
Metastatic disease?		no	no	yes	no	no	no	no	no
Reason for initiating treatment		progressive advanced **RCC** requiring surgery after unilateral nephrectomy, with a high probability of requiring kidney replacement therapy after surgery	patient refusal for further abdominal surgeries for **RCC**	metastasized **NET** with non-response to standard treatment	progressive advanced **retinal HBL** with threatened central vision on only eye	progressive advanced **RCC** requiring surgery after unilateral nephrectomy, with a high probability of requiring kidney replacement therapy after surgery and progressive central **retinal HBL**	progressive advanced bilateral **RCC** requiring surgery, with a high probability of requiring kidney replacement therapy after surgery	progressive advanced **RCC** requiring surgery after unilateral nephrectomy, with a high probability of requiring kidney replacement therapy after surgery	progressive advanced **retinal HBL** with threatened central vision on only eye
Age at treatment beginning		39	29	36	59	56	44	46	52

Belzutifan treatment duration	7,4 months (ongoing)	26 months (ongoing)	24,4 months (ongoing)	13,4 months (ongoing)	16,1 months (ongoing)	24,6 months (ongoing)	15,4 months (ongoing)	3,7 months (ongoing)
Best response to treatment (Relative tumor reduction calculations were based on reduction in largest lesion diameter)	**CNS HBL** (-19% at 5,6 months), **RCC** (-22% at 2,9 months)	**RCC** (-38 % at 20,7 months), **CNS HBL** (small lesions constant in number and size)	**gastric NET** (-74 % at 18 months), **pancreatic NET** (-59 % at 18 months), **hepatic metastasis of NET** (-50 % at 15 months), **CNS HBL** (-40 % at 24,3 months)	**pancreatic NET** (-28% at 6 months), **retinal HBL** (good clinical response with fibrotic and inactive tumor at 9,5 months)	**RCC** (-26 % at 11,3 months), **retinal HBL** (-50 % at 3,2 months), **CNS HBL** (small lesions constant in number and size)	**RCC** (-19 % at 24 months), **CNS HBL** (-20 % at 5,8 months)	**RCC** (-27 % at 14,9 months), **pancreatic NET** (-24 % at 11,6 months), **CNS HBL** (-30 % at 3 months)	**RCC** (-15 % at 3,3 months), **retinal HBL** (- 2 % at 3,3 months), **CNS HBL** (+25% at 3,3 months)
Tumor reduction procedures during Belzutifan treatment?	0	1 for new small **RA** treated with focal laser coagulation at 23,6 months	0	0	0	0	1 for recurrence of **RCC** at the nephrectomy site at 11,5 months	0
Adverse events (anemia and ALT or AST elevation)	Moderate anemia	Mild anemia	Mild anemia, liver enzyme elevation (peaked after 5 weeks of treatment)	Moderate anemia, liver enzyme elevation (peaked after 4 weeks of treatment)	Moderate anemia, liver enzyme elevation (peaked after 8 weeks of treatment)	Mild anemia and persistent mild respiratory infection of the upper respiratory tract	Mild anemia and herpes zoster infection on the arm	Moderate anemia, liver enzyme elevation (peaked after 3,6 months of treatment)
Treatment modifications	**Dose reduction** to 80 mg (due to anemia) after 2,1 months, **treatment interruption** for 2 weeks (due to stay abroad) and **ESA initiation** (due to anemia) after 4,4 months, **dose reduction** to 40 mg (due to anemia) after 6,9 months.	none	none	**Dose reduction** to 80 mg and **ESA initiation** (due to anemia) after 4,3 months	**Dose reduction** to 80 mg (due to anemia) at 3,5 months	**Dose reduction** to 80 mg (due to respiratory infection) for 4,6 months, **Treatment interruption** for 1 month (due to re-approval delay by insurer)	**Treatment interruption** for 1 week (due to herpes zoster infection) after 1 week of belzutifan, **treatment interruption** for 1 month (due to elective surgery with mild anemia) at 11 months.	**Dose reduction** to 80 mg and **ESA initiation** (due to anemia) after 2 months
