# Supplementary material for: Belzutifan for patients with Von Hippel-Lindau (VHL) disease-associated heterogeneous tumors – a retrospective single center analysis
Source: BMC Cancer. 2025 Nov 1;25:1686. doi: 10.1186/s12885-025-15192-8 (PMC12579424; doi:10.1186/s12885-025-15192-8)
Supplement: Supplementary file 3 — Supplementary Material 3. [file 12885_2025_15192_MOESM3_ESM.pdf]

Supplementary Table 2: Relative tumor reduction rate and tumor response rate under belzutifan treatment.

**Organ manifestation** (n= patients with measurable target lesions)	**Best relative tumor reduction rate (median in %)[1]** *in the first 12 months*	**Patients achieving SD or PR (in %)[2]**
RCC (n=6)	-21,5	86
CNS HBL (n=5)	-9	100
Pancreatic NET (n=3)	-28	100
Retinal HBL (n=2)	-28,2	75
Gastric NET (n=1)	-72,3	100
Hepatic metastasis (n=1)	+10	100

[1] *in measured target lesions*

[2] *in target and non-target lesions*

Stable disease (SD) and partial response (PR)
